# Virulent and attenuated strains of *Trichoderma citrinoviride* mediated resistance and biological control mechanism in tomato

**DOI:** 10.3389/fpls.2023.1179605

**Published:** 2023-05-30

**Authors:** Di Zhao, Dan Shen, Haiyan Fan, Xiaofeng Zhu, Yuanyuan Wang, Yuxi Duan, Lijie Chen

**Affiliations:** ^1^ Analysis and Testing Center, Shenyang Agricultural University, Shenyang, China; ^2^ College of Plant Protection, Shenyang Agricultural University, Shenyang, China; ^3^ College of Life Science and Technology, Shenyang Agricultural University, Shenyang, China

**Keywords:** *Trichoderma citrinoviride*, virulent strain, attenuated strain, mediated resistance, biocontrol mechanism

## Abstract

**Introduction:**

Root-knot nematode disease is one of the world's most serious vegetable crop diseases. In recent years, *Trichoderma* spp. has been widely used in root-knot nematode disease control as a biological control agent.

**Methods:**

Virulent and attenuated strains of *Trichoderma citrinoviride* mediated resistance and biological control mechanism in tomato were determined.

**Results:**

Preliminary experiments found differences in nematicidal virulence among *Trichoderma citrinoviride*. The 24-hour corrected mortality rate of the virulent strainT1910 was as high as 92.37%, with an LC50 of 0.5585 against the second juveniles (J2s) of *Meloidogyne incognita*. And the attenuated strain TC9 was 23.01%, the LC50 was 2.0615, so the virulent strain T1910 had a more substantial effect on the J2s than the attenuated strain. We found that the strong virulent strain T1910 have a good control effect on M. incognita by the pot experiment of tomato than that of the attenuated virulent strain TC9,especially the J2 and J4 numbers were inhibited inside the root knots of tomato. Theinhibition rates of virulent strains reached 85.22% and 76.91%, followed by attenuatedstrain TC9, which were 63.16% and 59.17%, respectively. To reveal the differences intomato defense pathways induced by different virulent strains, qRT-PCR was further usedto detect changes in the expression of inducement-related genes. The results showed thatthe TC9 was significantly upregulated at 5dpi, LOX1, PR1, and PDF1.2. The PR5 gene ofthe virulent strain T1910 was highly upregulated, and the JA pathway was activated laterbut weaker than the attenuated strain. The results of this study revealed that thebiocontrol mechanism of *T. citrinoviride* as poison killing through the virulent strain T1910 and induced resistance to *M. incognita* through attenuated strain, although virulence degradation also has an induced resistance effect. Moreover, the attenuated strain TC9 stimulated tomato immune response earlier than the virulent strain by nematode-associated molecular pattern-triggered (NAMP).

**Discussion:**

Therefore, the research elucidated the mechanism of multiple control of *Trichoderma* spp. against *M. incognita*.

## 1 Introduction

Plant parasitic nematodes are one of the major biotic stress factors wreaking havoc on agricultural and horticultural crops around the world, resulting in a dramatic drop in vegetable crop production. Diseases caused by plant pathogenic nematodes (PPN) have gradually become the second major category of diseases in agricultural production, among them, *M. incognita* is widely studied species with significant importance in reducing the yield of vegetable crops (Abd-Elgawad and Askary, 2018). Notably, these nematodes can benefit from parasitism in various crops such as grain, oil, vegetables, and fruits. It is estimated that the worldwide average crop loss due to pathogenic nematodes is 12.6% annually, equivalent to $215.77 billion (Reynolds et al., 2011). Due to the severe environmental pollution caused by chemical insecticides, the research and development of efficient and environmentally friendly biogenic insecticides have become a significant research focus (Chen and Song, 2021; Xie et al., 2022). *Trichoderma* spp. is an important biocontrol fungus that has been successfully applied to control many nematode diseases. Its biocontrol mechanisms include competition, hyperparasitism, induced resistance, and antibiotics, and almost all of them may be involved in the biocontrol process of nematodes (Contreras-Cornejo et al., 2015; Kumar and Khurana, 2021). Substances such as cellular hydrolases, antibiotics, vitamins, polysaccharides, and organic acids in *Trichoderma* fermentation fluid are high toxicity to nematodes. Importantly, they can destroy nematode cells through different mechanisms, or other metabolites enter nematodes, such as alkaloids that can inhibit necessary enzymes or act as DNA embedding agents (Tanaka et al., 2007). Notably, some flavonoids condense soluble cellular proteins, including essential enzymes, by forming complexes or rupturing cell membranes to inhibit nucleic acid synthesis (Plaper et al., 2003; Tsuchiya, 2015).

The biological control mechanisms of *Trichoderma* spp. are slightly different between the plants. Studies have shown that plants induce nematode-associated molecular pattern-triggered (NAMP) immunity in response to nematode infection through ROS, MAPK, jasmonic acid (JA), and salicylic acid (SA) signaling pathways (Fujimoto et al., 2011; Molinari et al., 2014; Fan et al., 2015; Sidonskaya et al., 2016; Song et al., 2017). Additionally, *Trichoderma* spp. can activate the plant defense system by releasing an activator that triggers the activation pathways of plant transcription factors and the expression of genes related to biotic and abiotic stress resistance (Shoresh et al., 2010; Sikora et al., 2014). *Trichoderma* spp. can also trigger SAR-related biochemical pathways and activate the expression of SA-dependent disease-related proteins, including PR-1, PR-5, and NPR1(Alfano et al., 2007; Arie et al., 2010; Mathys et al., 2012; Yoshioka et al., 2012). Furthermore, a unique pathway by which *Trichoderma* spp. colonizes plant roots to induce defense responses is based on the plant hormones jasmonate (JA) and ethylene (ET) as signaling molecules (Martínez-Medina et al., 2017). It has also been suggested that *Trichoderma* spp. induces reactive oxygen species (RBOH1) production, and this may be a significant defense strategy during plant growth after nematode invasion (Medeiros et al., 2017). However, how *Trichoderma* spp. regulate these hormones and other signaling pathways to exhibit inhibitory effects on root-knot nematodes has not been thoroughly studied.

Our team previously screened *T.citrinoviride* Snef1910 from 890 fungal strains and found it had a good biocontrol effect in field experiments. *T.citrinoviride* Snef1910 showed high virulence against J2s of *M. incognita*. Furthermore, *T. citrinoviride* Snef1910 significantly inhibited egg hatching with the hatching inhibition percentages of 90.27, 77.50, and 67.06% at 48, 72, and 96 h after the treatment, respectively. In the field experiment, the biocontrol application showed that the control efficacy of *T. citrinoviride* Snef1910 against root-knot nematode was more than 50%. Meanwhile, *T. citrinoviride* Snef1910 increased the tomato plant biomass. Moreover, strain Snef1910 showed significant antagonistic activity *in vitro* towards other pathogens that caused plant diseases in wheat, cotton, melon and other plants (Fan et al., 2020). So, *T. citrinoviride* is one of the potential biological control agent against root-knot nematode, *M. incognita*. Therefore, this study elucidated the mechanism of *T.citrinoviride* against by investigating the nematocidal activity of virulent and attenuated strains of *T.citrinoviride*, pot experiments and the expression levels of marker genes of each plant defense response pathway detected by fluorescence quantitative PCR. In doing so, we aim to provide a theoretical basis for developing biocontrol agents of *T.citrinoviride* in the future.

## 2 Materials and methods

### 2.1 Collection and isolation of *Trichoderma citrinoviride* isolates

In the present study, the virulent strains T1910 and attenuated strains TC9 were isolated from the original strains of *T.citrinoviride* Snef1910 (Fan et al., 2020) by UV-chemical mutagenesis, which differed in virulence from *M. incognita*. The spore suspension of *T.citrinoviride* (1×10^7^) was sucked into the 90 mm petri dish and placed under the UV lamp that had been preheated for 30 minutes. After 3 minutes of irradiation, 4 mL of the spore suspension of the strain screened by UV mutagenesis was prepared for DES (1 ml + 0.5 ml absolute ethanol) mutagenesis and incubated at 180 rpm for 30 min with shaking. The reaction was terminated with 0.5 ml of 25% sodium thiosulfate (Na_2_S_2_O_3_), and the strains of *T.citrinoviride* solution was evenly coated on 2% water AGAR plates for cultivation. After the spores grew out, the subsequent tests were carried out (Zhao et al., 2016). All the above strains were stored in Nematology Institute of Northern China, Shenyang Agricultural University.

### 2.2 Reparation of liquid medium of *T.citrinoviride* isolates

*T. citrinoviride* spores and mycelia cultured on PDA plates were inoculated in a triangle flask containing PD liquid medium aseptically and incubated at 25°C and 120 RPM for 7 days by shaking. The mycelia and spores were removed by filtration with a filter device equipped with a 0.22 μm filter membrane to prepare the fermentation broth of different strains of *T.citrinoviride*. The liquid medium of *T.citrinoviride* spore was obtained as follows. First, 5 ml sterile water was added to the colonies, and the spores on the surface were washed off gently. Then, the spore suspension was put in 50 ml sterilized conical flask that had been placed with the sterile glass ball in advance, after sufficient oscillation with sterilized cotton wool to filter and sterile water flushing residue 2-3 times, eventually make the concentration of spore was 10^8^ cfu/ml (Zhao et al., 2016).

### 2.3 Nematode culture and infection assays

A pure culture of *M. incognita* was maintained in tobacco roots grown under pot culture conditions (28± 2°C) at the Institute of Northern Nematode, Shenyang Agricultural University. Second-stage juveniles (J2s) of *M. incognita* were inoculated into the one-month-old tomato seedlings, and regular watering was done to maintain the optimum moisture level for plant growth. Seedlings were uprooted 30 days post-inoculation of nematodes, and the roots were gently washed in tap water to remove the adhering soil particles. Egg masses of *M. incognita* were collected from the roots with the help of forceps under a stereo-zoom microscope. Gathered egg masses were placed in a modified Baermann funnel setup for 3 to 5 days to obtain the uniformly hatched J2s. Finally, after estimating the population density of these juveniles under a stereo-zoom microscope, they were used for further experiments (Zhao et al., 2016).

Morphologically and biochemically characterized *T.citrinoviride* isolates were tested for their *in vitro* efficacy against *M. incognita*. About 100 J2s of *M. incognita* were placed in sterile 24-well plates containing 200 μl fermentation broth of *T.citrinoviride*. And the liquid medium of *T.citrinoviride* isolates (10^8^ cfu/ml) were taken at the rate of 0.05 µl and mixed with 1 ml water and applied to the *M. incognita*. Each treatment was replicated 3 times in a completely randomized design (CRD). The 24-well plates were incubated at room temperature (28 ± 2°C), and observations on dead juveniles were recorded at 24 h under a stereo zoom microscope. The mortality rate and corrected mortality rate of nematodes were calculated. Furthermore, the NaOH stimulation method was used to determine the survival of J2 of *M. incognita*.


$$\text{Nematode mortality}/\text{\% }=\frac{\text{Number of dead nematodes}}{\text{Number of nematodes tested}}\times 100$$

$$Corrected\;mortality/\text{\% }=\frac{Treated\;nematode\;mortality\;-Control\;nematode\;mortality}{1\;-\;Control\;nematode\;mortality}\times 100$$

Approximately 4-week-old tomato seedlings were transplanted into the mud pots (15 cm dia × 30 cm height) filled with 1 kg of sterilized soil. After the establishment of seedlings in the pots, second-stage juveniles of *M. incognita* were inoculated around the plant’s root zone by removing the top soil layer. The temperature of the pot experiment was 25°C ± 3°C. Three days after inoculating nematodes, a fermentation broth of virulent and attenuated strains of *T.citrinoviride* isolates was applied to the rhizosphere region of the plant. Plants inoculated with nematode alone served as untreated control. Treatments were arranged in CRD with 3 replicates per treatment. Plants were watered regularly to the field capacity. Finally, 5, 10, and 15 dpi after nematode inoculation, plants were uprooted carefully and assessed for their growth parameters and the population of nematodes. The gall index is shown in **Table 1**. Then the root of tomato reactive oxygen species staining were evaluated. CMH_2_DCFDA (C6827) molecular probe was prepared with phosphate buffer (PB), and the root was incubated with CM-H_2_DCFDA (10 μM) and treated at 4°C for 90 min. The samples were washed with KCl (0.1 mM) and CaCl (0.1 mM) to remove excess CM-H_2_DCFDA. Samples were stored at room temperature for 1 h before being photographed with an Olympus C-5050 digital camera.

**Table 1:** The classification of the Gall index.

Gall index	Classification standard
Level 0	No root knots on all roots, no infection
Level 1	The number of root knots on the root system was 1%- 20%
Level 2	The number of root knots on the root system was 21% to 40%
Level 3	The number of root knots on the root system was 41% to 60%
Level 4	The number of root knots on the root system was 61% to 80%
Level 5	The number of root knots on the root system was 81-100%


$$\text{Control efficacy}/\text{\% }=\frac{\text{Control root knot index }-\text{ Treated root gall index}}{\text{Control root knot index}}\times 100$$

### 2.4 RNA isolation and quantitative real-time PCR

In order to analyze the differences in control effects of virulent and attenuated strains of *T.citrinoviride* against *M. incognita*, q-PCR was used to detect the expression of defense genes in tomato stimulated by *T.citrinoviride*. The expression levels of auxin related genes *ARF1*, reactive oxygen species related genes *RBOH1*, jasmonic acid related genes *LOX1*, salicylic acid related genes *PR1*, *PR5* and *NPR1* and jasmonic acid pathway related genes *PDF1.2* were detected to reveal the induction and resistance mechanism from the view of defense genes. To provide some basic data for the theoretical research on how *T. citrinoviride* regulate these hormones and other signaling pathways to exhibit inhibitory effects on root-knot nematodes in vegetables.

Primers sequence of tomato genes related to induction-resistance pathway of real-time fluorescence quantitative PCR were synthesized by Shanghai Shenggong Bioengineering Technology Co., LTD., and the length of the primers was 80-300 bp. In this study, gene-specific primers were designed according to the reference sequences of *ARF1*, *RBOH1*, *LOX1*, *PR1*, *PR5*, *NPR1*, and *PDF1.2* in NCBI which be stimulated by *T.citrinoviride*, as shown in **Table 2**.

**Table 2:** Primer sequences of genes related to inducing resistance pathway in tomato.

The name of the primer (Primer)	Genetic sequence (Gene sequence)
ARF1-F	GCAGCAACACCTACAAC
ARF1-R	ACAGGAGACTTCCACATTC
RBOH1-F	GTCAGGCTTCTACAGAAAAC
RBOH1-R	GTTGATTACAGTAGCCGGTTC
LOX1-F	GCCTCTCTTCTTGATGGA
LOX1-R	GTAGTGAGCCACTTCTCCAA
PR1-F	CCTCAAGATTATCTTAACGCTC
PR1-R	TACCATTGCTTCTCATCAACC
PR5-F	CAACATCCCTATGTCTTTCGGC
PR5-R	AGGACCACATGGACCTTGAGTG
NPR1-F	GCGATATTCCAACCTATA
NPR1-R	TAGATTCAAATACACCATTC
PDF1.2 -f	AAAAAGTGGCAAGTGGAATGG
PDF1.2 -R	AATGGCAAGGTGAGTAGCAGTAA
Actin-F	CACCACTGCTGAACGGGAA
Actin-R	GGAGCTGCTCCTGGCACTTT

The J2s of *M. incognita* were inoculated around the root zone of the tomato and treated with sterile water (CK+ RKN), fermentation broth of virulent strains (T1910 + RKN), and fermentation broth of attenuated strain (TC9 + RKN). At 5, 10, and 15 dpi, the whole tomato roots were washed with water, dried with filter paper, labeled, and stored in a refrigerator at -20°C until used. Three tomato plants were treated as one treatment, each repeated three times.

qRT-PCR analysis of nematode resistance gene. Total RNA was extracted from tomato roots (four biological replicates per treatment) using HiScript^®^ II Q Select RT SuperMix for qPCR (+ gDNA wiper) (Nanjing Norvezan Biotechnology Co., LTD.). The cDNA was constructed from 1µg RNA of each sample after treatment with RNase DNase I (Kangwei Century Company) using the HiScript III 1st Strand cDNA Synthesis Kit (+gDNA Wiper) (Nanjing Norvezan Biotechnology Co., LTD.) along with the manufacturer’s instructions(CW0588S). The reaction was performed in 25 μL containing 1 μL cDNA (10 ng), 0.5 μL of each specific primer (10 pM) (previously designed by Abbasi et al. (Abbasi et al., 2019). The reaction program including 95° C for 3 min followed by 40 cycles (95° C/30 s, 55° C/45 s, and 72° C/30 s), with the final extension step (72° C/3 min). The results were presented as a relative increase or decrease in the expression of the target gene relative to the internal gene (*TUB*). Changes were calculated using REST software(Pfaffl et al., 2002). The results were determined by using the 2^-ΔΔCt^ method (Schmittgen and Livak, 2008). The correctness of the amplified products was judged by the melting curve. Furthermore, the samples of each treatment were repeated three times.

### 2.5 Data analysis

IBM SPSS Statistics 25.0 software was used for statistical analysis. Data were analysed on absolute values using ANOVA to test the efficiency of *T.citrinoviride* isolates against *M. incognita* population tested *in vitro*, pot culture and field studies. The means were analysed by Least Square Difference (LSD) and separated using Tukey’s HSD test at P≤ 0.001.

## 3 Results and analysis

### 3.1 *In vitro* effect of *T.citrinoviride* strains against *M. incognita* juveniles

**Figure 1:**
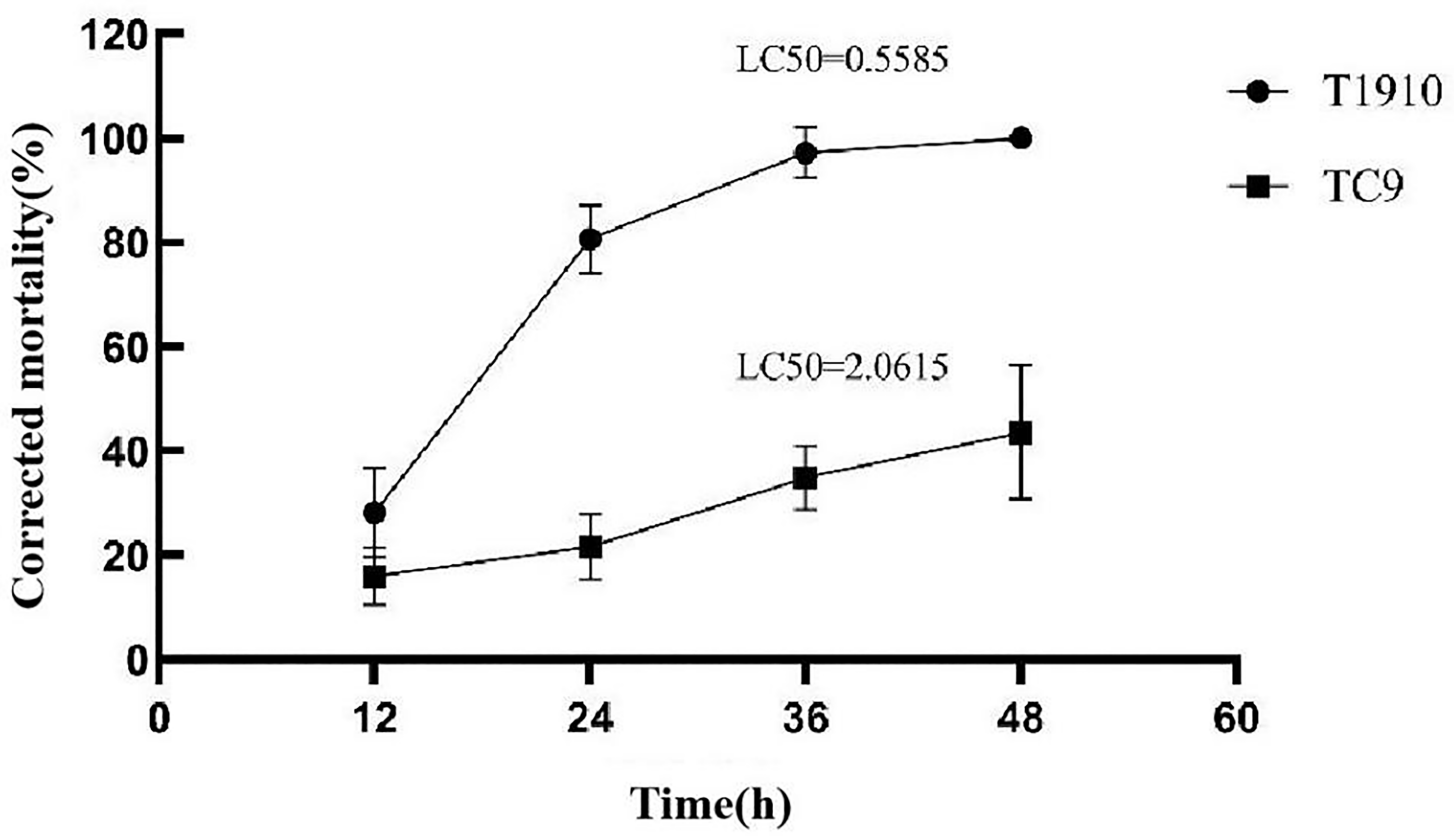
The corrected mortality rate and the LC50 of virulent strains T1910 and attenuating strain TC9 to *M. incognita* J2 at different times.

Our team previously screened *T.citrinoviride* Snef1910 from 890 fungal strains and found it had a good biocontrol effect in field experiments (Fan et al., 2020). However, the effect of *T.citrinoviride* strains against J2 were found to be unstable in the process of in-depth research. *T.citrinoviride* strain T1910 showed high activity against J2. The corrected mortality rate reached 80.71% at 24 h after treatment and 100% at 48 h with increased time (LC50 = 0.5585). However, the activity of the attenuating strain TC9 against J2 was low, and the corrected mortality rate was only 21.26% at 24 h and 43.61% at 48 h, with LC50 = 2.0615. In this study, two strains of *T.citrinoviride* screened by UV-chemical mutagenesis showed significant differences in toxicity against J2, which were named virulent strain T1910 and attenuated strain TC9. Additionally, these strains were used to further study the biological control mechanisms of *T.citrinoviride* (**Figure 1**).

**Figure 2:**
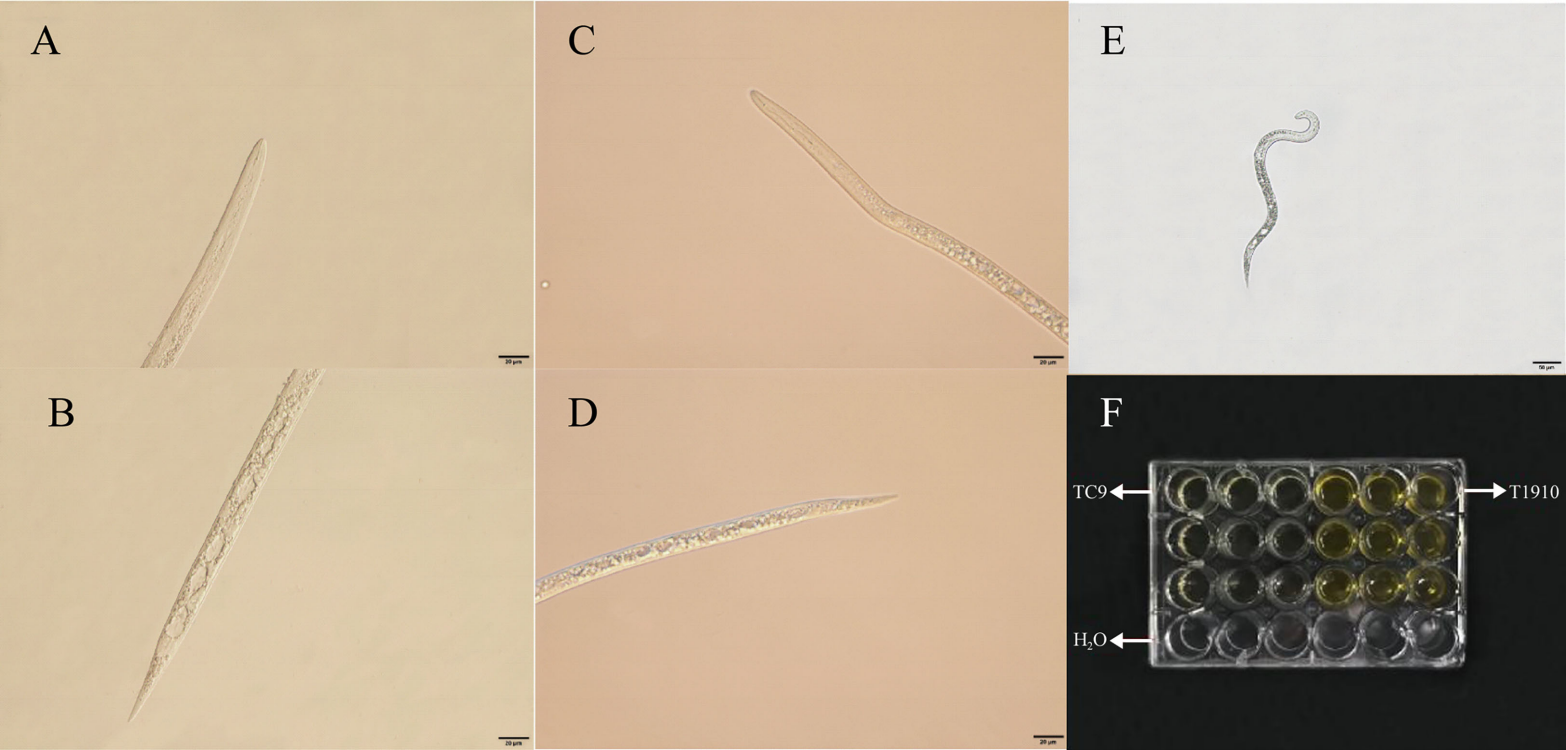
The *M. incognita* J2 died under the treatment of *T.citrinovirid*e. **(A, B)** The throat and middle and back of the body of *M. incognita* treated with T1910. **(C, D)** The throat and the middle and back of the body of *M. incognita* treated with TC9. **(E)** The *M. incognita* J2 died under H_2_O treatment. **(F)** Contact test of each treatment.

Using light microscopy, we observed that the anatomy of the J2 of *M. incognita* was disrupted following different strains (**Figure 2**). The results of the microscopy indicated that the nematode was in a good state in sterile water at 48 h. Still, in the fermentation broth of virulent strain T1910, the target nematode J2 was rigid. The body was chaotic, organs were decomposed, body walls were leaked, and serious vacuolation was observed in the body. However, the target nematodes died in the fermentation broth of the attenuated strain TC9. Still, there was no apparent decomposition in the worm body, and the nematodes’ surface structure, cuticle, and coelom were intact.

**Figure 3:**
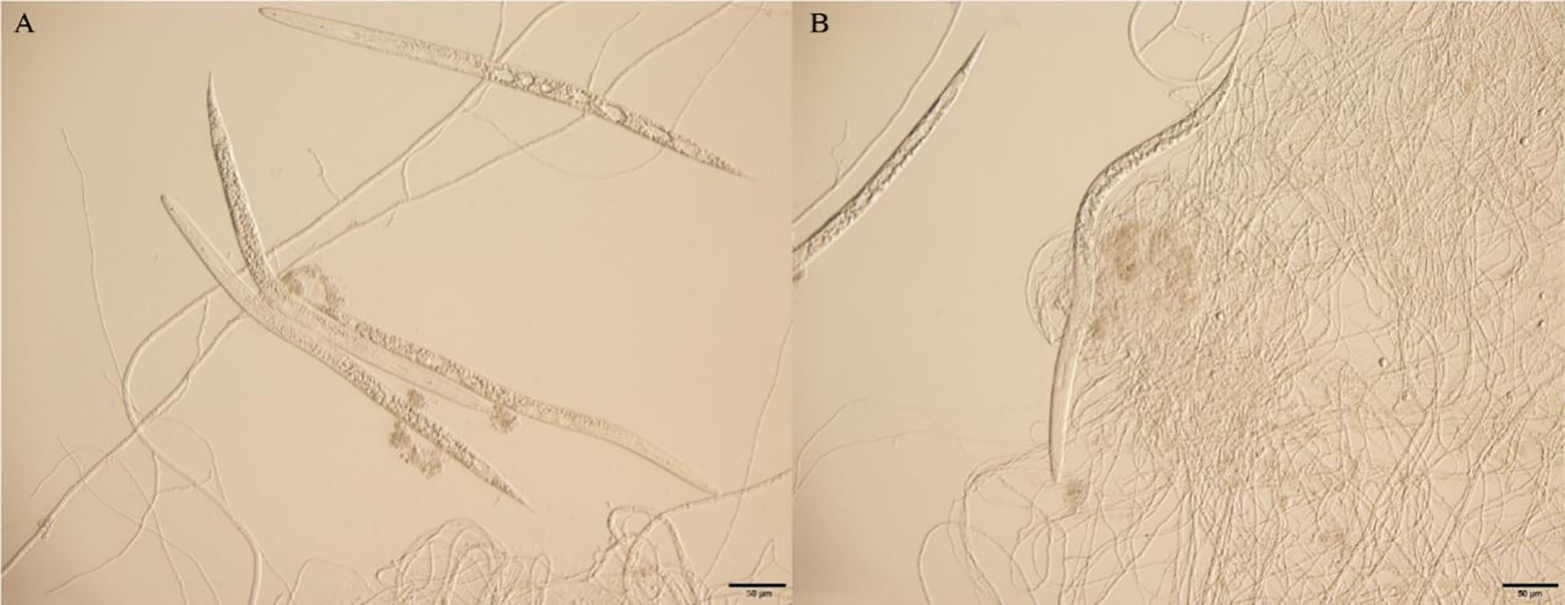
The effect of J2 of *M. incognita* being hyperparasitismed by virulent strain T1910 and attenuated strain TC9, after 24 h at 25°C. **(A)** The J2 of *M. incognita* was observed to be entangled and heavily parasitized by mycelium treated with the spore suspension of virulent strain T1910. **(B)** A few of the J2 of *M. incognita* treated with the conidial suspension of the attenuated strain TC9 were trapped by mycelia, unable to move, but still survived, and no bubbles were formed in the body.

After 24 h at 25°C, the J2 of *M. incognita* was observed to be entangled and heavily parasitized by mycelium treated with the spore suspension of virulent strain T1910. The worm’s body became rigid and lost vitality. The body wall was destroyed with many bubbles in the body, and the contents leaked. A few of the target nematode J2 worms treated with the conidial suspension of the attenuated strain TC9 were trapped by mycelia, unable to move, but still survived, and no bubbles were formed in the body (**Figure 3**).

### 3.2 Effect of virulent and attenuated strain of *T.citrinoviride* on *M. incognita* population and growth of tomato plants under pot culture conditions

**Figure 4:**
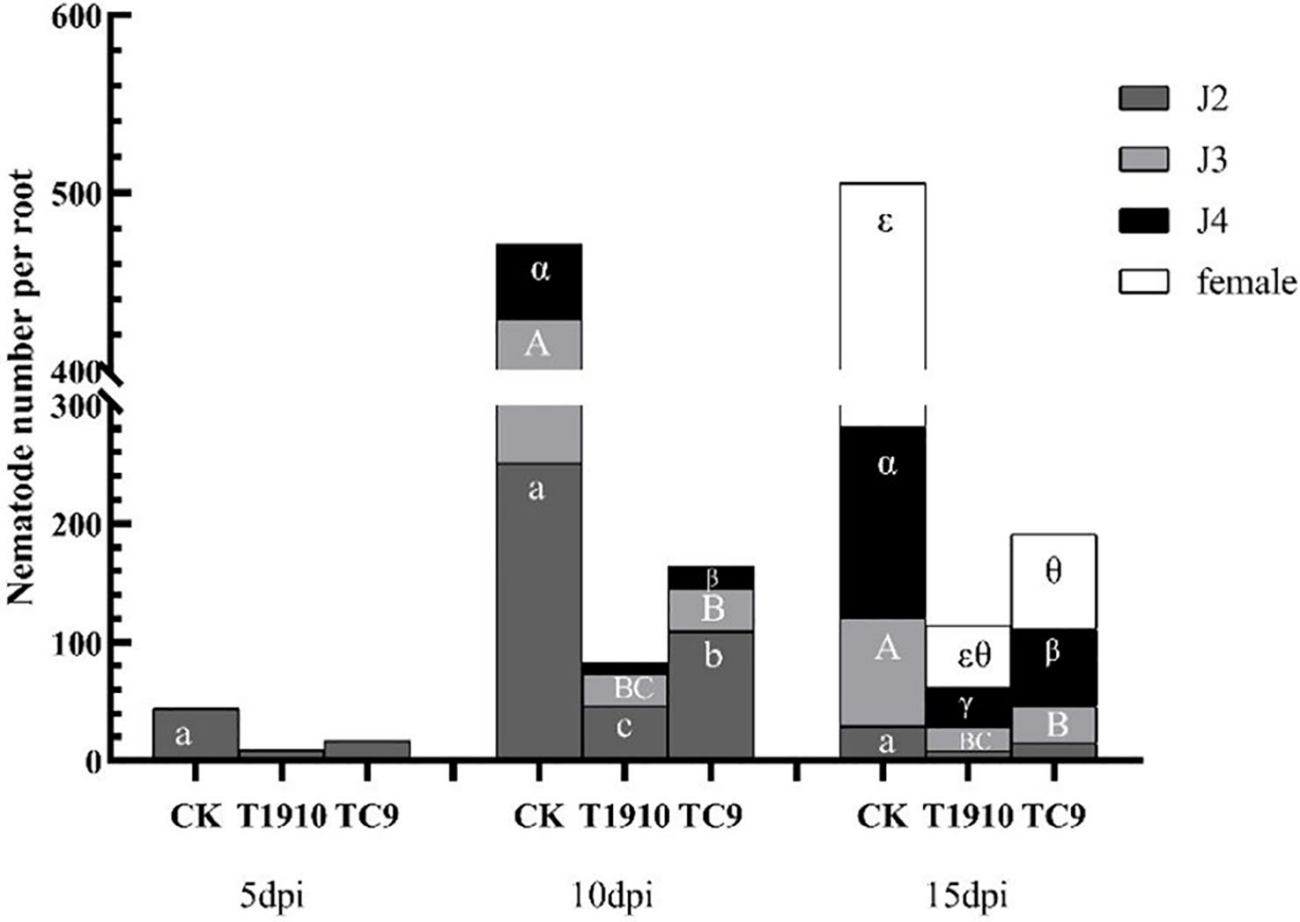
The number of root-knot nematodes in different stages in tomato roots treated with different virulent strains at different times. Different letters indicate significant differences (T-test with P<0.05): a or b indicates the significant differences in the J2 stage; A or B indicates the significant difference in the J3 stage; α or β indicates the significant difference in the J4 stage; ϵ or θ indicates the significant difference in adult females. Same as below females.

**Figure 5:**
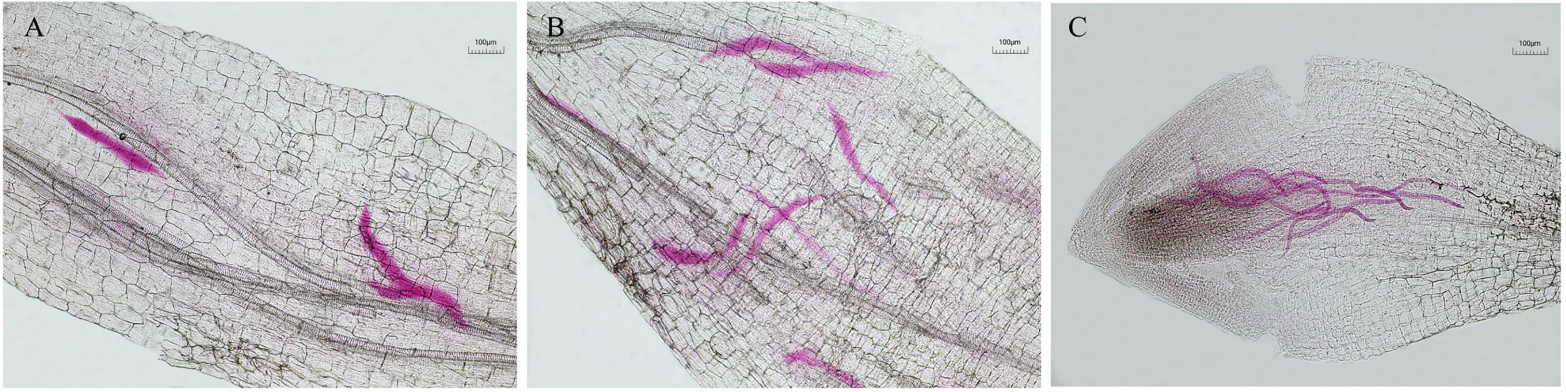
Nematode infection in tomato root system under each treatment at 5dpi. **(A)** virulent strains T1910 **(B)** attenuating strain TC9 **(C)** H_2_O.

The pot experiment results showed that compared with the control, the number of nematodes in the tomato root system was significantly reduced at 10 dpi and 15 dpi by virulent and attenuated strains of *T.citrinoviride*. The inhibition rates of virulent strains reached 85.22% and 76.91%, respectively, followed by attenuated strain TC9, which were 63.16% and 59.17%, respectively. As root-knot nematodes developed from J2 to J3, J4, and adult, both virulent and attenuated strains could significantly inhibit the infection and development of nematodes on tomato roots. Compared with the attenuated strain TC9, the virulent strain T1910 significantly inhibited the invasion of J2 and the development of J4 (**Figures 4**, **5**).

**Figure 6:**
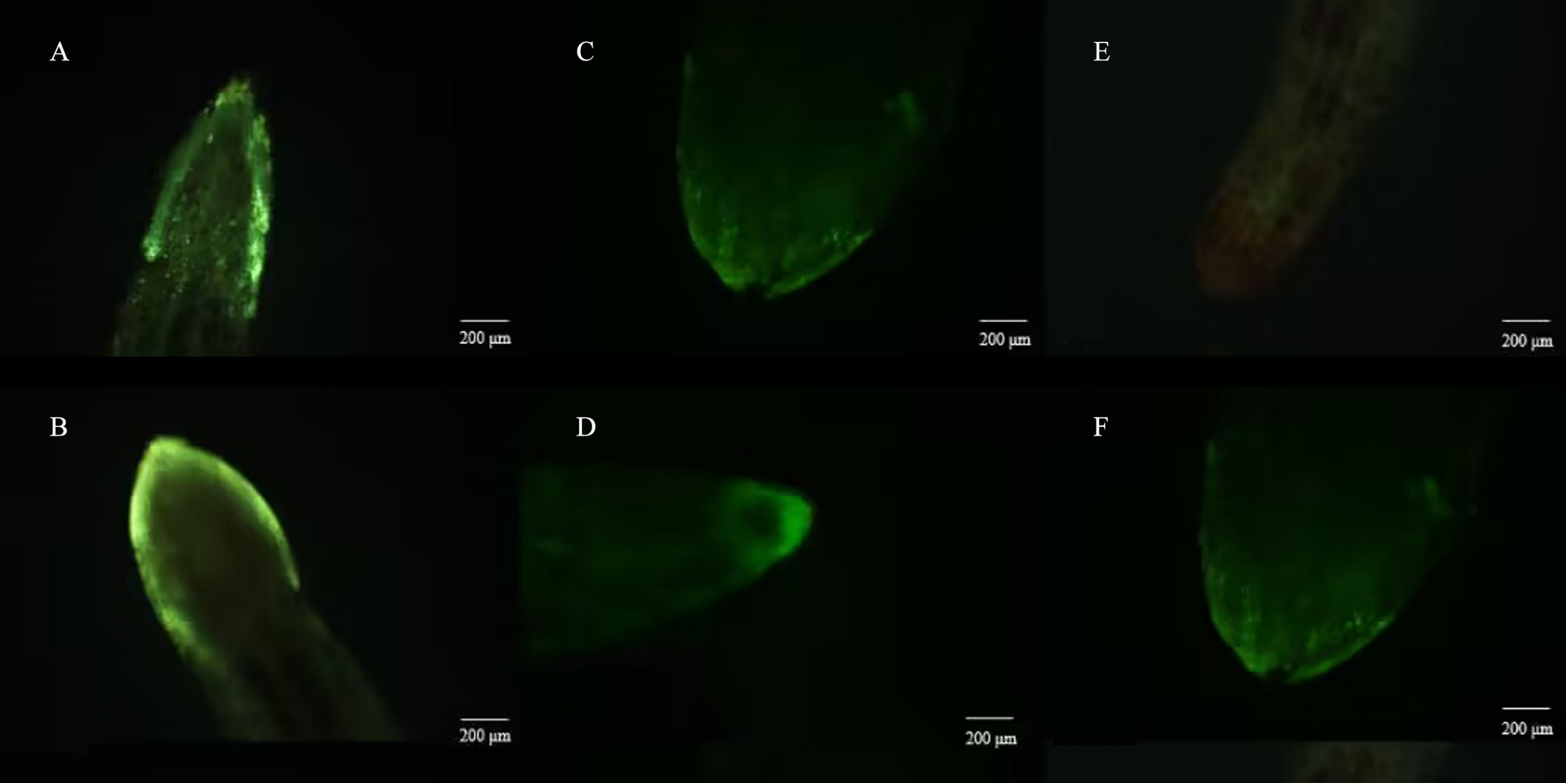
Reactive oxygen fluorescence staining of tomato roots after be treated by *T.citrinoviride*. **(A)** and **(B)** virulent strains T1910. **(C)** and **(D)** attenuating strain TC9. **(E)** and **(F)** H_2_O.

Meanwhile, the reactive oxygen species staining assay showed that virulent strain T1910 could activate the burst of reactive oxygen species in plant roots more than attenuated strain TC9 (**Figure 6**).

To further verify the control effect of virulent and attenuated strains on southern root-knot nematodes, the changes in the number of root knots of nematodes were measured in a tomato pot experiment. Compared with the control group, the number of root knots of tomato roots was significantly reduced by 10 dpi and 15 dpi treatment with virulent strain T1910, and the control effect was 76.59% and 77.39%, respectively. However, treatment with attenuated strain TC9 also reduced root-knot formation. However, this effect was insignificant (**Table 3**). The experiment result shows that even though the virulence of *T.citrinoviride* was degraded, the immune stimulation and growth promotion effects still significant by the research of induce resistance through fermentation broth to stimulate the immune pathway of tomato to resist *M. incognita* infection.

**Table 3:** Gall index and control efficiency of *T.citrinoviride* strains.

Treatments	Gall index (0~5)/Times			Control efficiency(%)/Times		
	5 dpi	10 dpi	15 dpi	5 dpi	10 dpi	15 dpi
T1910	0.1 ± 0.06B	0.2 ± 0.07B	0.9 ± 0.22B	84.91 ± 10.39a	76.59 ± 6.68a	77.39 ± 6.15a
TC9	0.2 ± 0.03B	0.6 ± 0.15B	1.9 ± 0.12AB	64.15 ± 7.55b	36.79 ± 15.05b	47.68 ± 12.49b
CK	0.5 ± 0.21B	1.0 ± 0.58AB	3.7 ± 0.80A	——	——	——

### 3.3 Expression analysis of genes related to the resistance of tomato to *M. incognita* induced by virulent and attenuated strains of *T.citrinovirid*e

Results of the study revealed that the cell free culture filtrate of virulent and attenuated strains enhanced the mortality of *M. incognita* juveniles. Among the different isolates tested, the attenuated strains of *T.citrinoviride* TC9 at its 100 per cent concentration recorded about 43.61% of juvenile mortality after an exposure period of 48 h. It has been evident from the present study that there are other bio-control mechanisms. Therefore, the gene expression analysis of nematode resistance induced by virulent and attenuated strains of *T.citrinoviride* in tomato have been further studied. Notably, the expression of auxin-related gene *ARF1*, reactive oxygen species-related gene *RBOH1*, ester oxygenase related gene *LOX1*, salicylic acid-related genes *PR1*, *PR5*, *NPR1*, and jasmonic acid-related gene *PDF1.2* was further measured to explore the biological control mechanism of *T.citrinovirid*e induced tomato against *M. incognita*.

**Figure 7:**
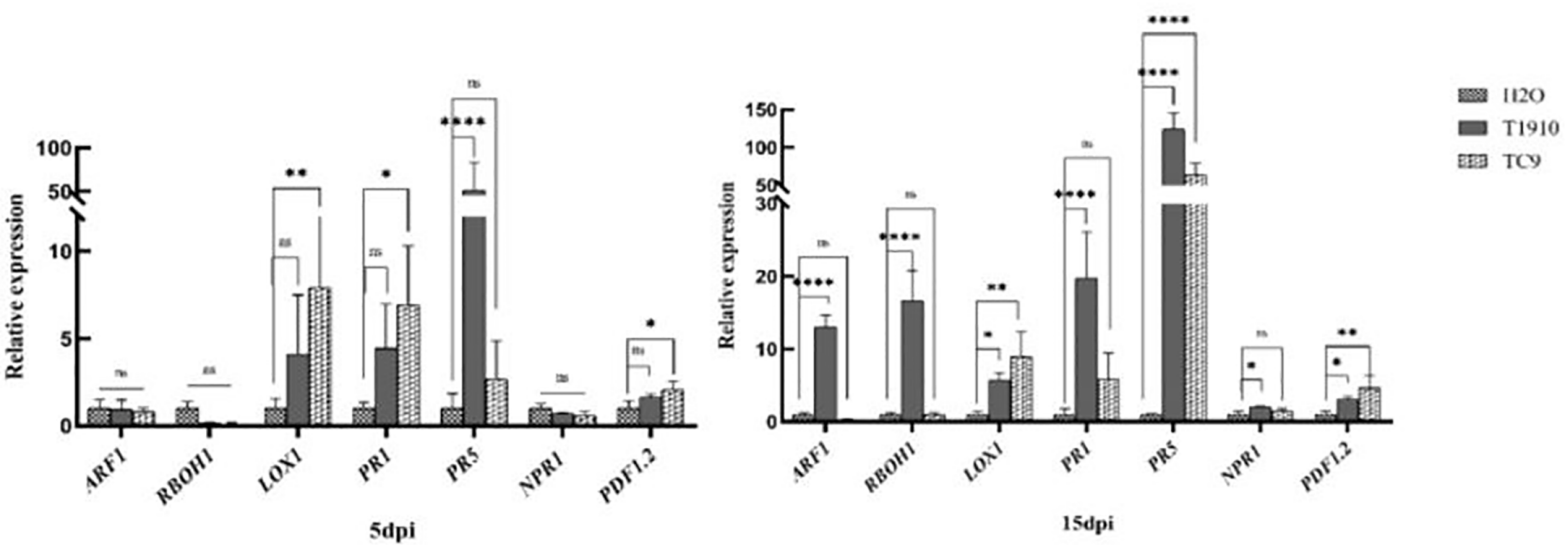
Relative gene expression in response to induced by T1910 and TC9. The asterisk (*), (**), (****) indicate P < 0.05, P < 0.01, and P < 0.0001 (t-test) for each treatment versus control (five replicates). And the expanded form of "ns" is “not statistically”.

At 5 dpi, only the *PR5* gene was significantly upregulated in virulent strain T1910, which was 51.6 times higher than that of the control, while *LOX1*, *PR1*, and *PDF1.2* genes were significantly upregulated in attenuated strain TC9. At 15 dpi, *ARF1*, *RBOH1*, *LOX1*, *PR1*, *PR5*, *NPR1*, and *PDF1.2* genes of virulent strain T1910 were significantly upregulated, and *PR5* was still the highest, which was 123.9 times of the control. *LOX1* and *PDF1.2* genes of virulent strain TC9 were still significantly upregulated, but *PR1* was not significantly upregulated. However, *PR5* was significantly upregulated up to 62.8 times (**Figure 7**). Our research suggests that the attenuated strain TC9 stimulated both the SA and JA signaling pathways at the early stage of inoculation, thus inhibiting the infection and development of nematodes. In contrast, the virulent strain only stimulated the SA signaling pathway at the early stage and the JA pathway at the later stage.

## 4 Discussion

The current study demonstrated the *in vitro* mortality rate of *M. incognita* juveniles using two strains of *T.citrinoviride*. It was found that the fermentation broth of the virulent strain T1910 was the most active against J2 of *M.incognita*., while the fermentation broth of the attenuated strain TC9 was less virulent. In this process, it was found that the body wall of the poisoned nematodes was thinner, and bubbles were formed in the body. These phenomena may result from the degradation of the body wall by protease or chitinase in the fermentation broth or the contact of metabolites in the fermentation broth. In addition, when nematodes were wounded by mycelium, the body wall leaked, which was the function of heavy parasitism and secondary metabolites. Hadavi (2008) found that hydrolases produced by *Trichoderma* could inhibit nematodes and eggs to varying degrees. Jiao et al. (2015) found that two strains of *T. harzianum* were beter than *T. viride* in their activity to *M.incognita*. Also, the cuticle of juveniles and shell of eggs were digested eggs hatching delayed, and the intestinal cavity of larvae showed vacuolation. Likewise, the pot experiment investigated the biocontrol effect of *T.citrinoviride* on *M. incognita*. Both virulent strain T1910 and attenuated strain TC9, showed significant growth-promoting effects on tomatoes and inhibited the number of root knots. The attenuated strain TC9 had the most significant growth-promoting effect, and T1910 had an optimal ability to reduce the number of root knots. The results of reactive oxygen species fluorescence labeling staining showed that both wild-type and attenuated strains could stimulate the burst of reactive oxygen species, and virulent strain T1910 exhibited more robust activation activity and brighter fluorescence than attenuated strain TC9. Therefore, these results indicate that different virulence strains of *T.citrinoviride* play a particular role in the biological control of nematode diseases. However, the modes of action may differ, and it has the potential for biological control.

The biocontrol mechanisms of *T.citrinoviride* include competition, hyperparasitism, induced resistance, and antibiotics, and almost all of them may be involved in the biocontrol of nematodes (Contreras-Cornejo et al., 2015). Substances such as cell hydrolases, antibiotics, vitamins, polysaccharides, and organic acids in *Trichoderma* fermentation fluid are highly virous to nematodes. They can destroy nematode cells through different mechanisms or use their metabolites to enter nematodes, such as alkaloids that can inhibit necessary enzymes or act as DNA embedding agents(Tanaka et al., 2007). Some flavonoids condense soluble cellular proteins, including essential enzymes, by forming complexes or rupturing cell membranes to inhibit nucleic acid synthesis (Tariqjaveed et al., 2021). Based on different virulence strains of *T.citrinoviride*, the nematocidal activity, hyperparasitism activity, growth promotion, and inducement and resistance of the two strains were detected, and the biocontrol mechanism was studied from multiple perspectives.

In the gene expression tests, it was found that the virulent strain T1910 induced *PR1* at the early stage of nematode infection (5dpi), and the root tissues of tomato provided a rapid SA-regulated defense response to protect the roots from RKN invasion (Martínez-Medina et al., 2017). In the late stage (15dpi), auxin was significantly activated, including reactive oxygen species, *LOX1*, *PR1*, *PR5*, and *NPR1* markers of the SA pathway, and the PDF1.2 gene related to the JA pathway. The accumulation of auxin was associated with controlling plant growth and related to the change in cell REDOX status, including the activation of NADPH oxidase and cell wall peroxidase to generate oxidative bursts. Simultaneously, the high expression of ester oxygenase caused the peroxidation of membrane lipids and induced the burst of reactive oxygen species to provide the appropriate defense response (Considine and Foyer, 2014). These results indicated that the JA-regulated defense pathway of virulent strains was not induced at the early stage of nematode development. Additionally, the virulent strains caused SA, JA pathways, and reactive oxygen species to resist nematode reproduction and secondary invasion during the breeding period.

The attenuated strain TC9 activated ester oxygenase *LOX1*, *SA* gene *PR1*, and JA pathway-related gene PDF1.2 at the infection-development stage (5dpi) and strongly induced *LOX1*, *PR5* and *PDF1.2* at the breeding stage (15dpi). The high expression of *PR1 and PR5* indicated that the attenuated strain also induced SA defense in plants and regulated the JA defense pathway through a lipid oxidation reaction. This phenomenon showed that the attenuated strain induced and successfully stimulated a strong ISR response. Furthermore, its protective effect depended on enhancing the JA defense mechanism against nematode invasion. Therefore, the JA pathway was the primary defense strategy of attenuated strain against RKN (Selim et al., 2014).

The attenuated strain TC9 of *Trichoderma* spp. had a weakened killing effect on J2 of *M. incognita*, but it still had a field control effect on *M. incognita* in tomato. However, the results show that the mechanism is still unclear by promoting tomato growth and enhancing immunity against nematode infection. Importantly, this study explored from the viewpoint of defense gene expression and found that the attenuated strain significantly stimulated the JA pathway. Therefore, the JA pathway may regulate systemic resistance in tomatoes, but the specific regulatory mechanism needs further study.

Root-knot nematode is one of the most significant diseases of crops in the world, especially tomato and other vegetables are its main hosts. At present, the biological control is very promising to root-knot nematode. There are many kinds of *Trichoderma* spp. in the world, which may also antagonize nematodes by complex and diverse mechanisms except competition. The application of *Trichoderma* spp. has been found effective in reducing root-knot nematode populations infesting vegetable crops (Khan et al., 2018). In this study, the potential mechanism of *T.citrinoviride* in the prevention and control of root-knot nematode was studied by evaluation of J2s efficacy *in vitro*, pot culture and expression analysis of genes. *T. citrinoviride* could be used as a potential biological control agent against root-knot nematode, *M. incognita*.

## Data availability statement

The original contributions presented in the study are included in the article/supplementary material. Further inquiries can be directed to the corresponding author.

## Author contributions

DZ: Methodology, Data analysis and Writing. DS: Provision of study materials. HF: Validation. XZ: Data Curation. YW: Formal analysis. YD: Review and Editing. LC: Writing, Conceptualization and Funding acquisition. All authors contributed to the article and approved the submitted version.
